# Towards a Long-Term Strategy for Voluntary-Based Internal Radiation Contamination Monitoring: Representativeness of the Monitoring Results in Fukushima, Japan

**DOI:** 10.3390/ijerph14060656

**Published:** 2017-06-20

**Authors:** Shuhei Nomura, Masaharu Tsubokura, Michio Murakami, Kyoko Ono, Yoshitaka Nishikawa, Tomoyoshi Oikawa

**Affiliations:** 1 Department of Global Health Policy, Graduate School of Medicine, The University of Tokyo, 7-3-1, Hongo, Bunkyo-ku, Tokyo 113-0033, Japan; 2 Department of Epidemiology and Biostatistics, School of Public Health, Imperial College London, Norfolk Place, London W2 1PG, UK; 3 Department of Radiation Protection, Minamisoma Municipal General Hospital, 2-54-6 Takami-cho, Haramachi-ku, Minamisoma, Fukushima 975-0033, Japan; tsubokura-tky@umin.ac.jp (M.T.); minamisoma-kyukyu@bz04.plala.or.jp (T.O.); 4 Department of Health Risk Communication, Fukushima Medical University School of Medicine, 1 Hikarigaoka, Fukushima, Fukushima 960-1295, Japan; michio@fmu.ac.jp; 5 Radiation Medical Science Center for the Fukushima Health Management Survey, Fukushima Medical University, 1 Hikarigaoka, Fukushima, Fukushima 960-1295, Japan; 6 Research Institute of Science for Safety & Sustainability, National Institute of Advanced Industrial Science and Technology, 16-1 Onogawa, Tsukuba, Ibaraki 305-8569, Japan; kyoko.ono@aist.go.jp; 7 Department of Health Informatics, School of Public Health, Kyoto University, Yoshida-Konoe, Sakyo-ku, Kyoto 606-8501, Japan; ynishikawa-tky@umin.ac.jp

**Keywords:** 2011 Fukushima nuclear incident of Japan, voluntary internal radiation monitoring program, representativeness

## Abstract

Following Japan’s 2011 Fukushima nuclear incident, voluntary participation, rather than mandatory, was adopted as the default scenario for individual radiation monitoring. We evaluated the representativeness of the internal monitoring results from voluntary participants in Minamisoma City, which is located 10–40 km from the Fukushima nuclear plant. Of approximately 70,000 individuals who were residing in Minamisoma City before the incident, a total of 19,263 residents (aged ≥21 years) participated in the monitoring from 1 October 2011 to 31 March 2015. Based on regression projection techniques with the available data obtained from the voluntary participants, the modeled probabilities of radiocesium (Cs) detection in October 2011 for Cs-137 and Cs-134 were 66.9% and 52.9%, respectively, which declined dramatically within a year following the incident. The rate of decline had stagnated since mid-2012, and the probability was close to zero after mid-2014. Sufficient agreement between the modeled probabilities of Cs detection (for the whole population) versus the measured Cs levels (for voluntary participants) was observed, except for Cs-134 in October 2011, indicating that the voluntary monitoring participant group was a good representative sample. Our findings affirmed the clinical importance of voluntary-based monitoring as a screening and dose-assessment tool in a post-nuclear incident. Our study informs societal decision-making regarding the long-term maintenance of the monitoring program under the current low exposure levels.

## 1. Introduction

On 11 March 2011, a massive earthquake and the resultant tsunami struck the northeast area of Japan, causing serious damage to the Fukushima Daiichi Nuclear Power Plant, and a large quantity of radioactive substances was discharged to the surrounding environment [1]. In response to major public concerns over radiation exposure in radiation-contaminated areas, government authorities, public utilities, and private companies initiated individual external and internal radiation monitoring to establish and implement a resilient radiation protection and safety program [2,3].

The purpose of individual external/internal radiation monitoring after a radiological incident consists of screening (or triage) and dose assessment [4]. The early response to a radiological incident is conducted in two phases, defined as follows: (1) the emergency phase is the beginning of a radiological incident for which immediate decisions for effective use of protective measures are required, such as evacuation and sheltering; and (2) the intermediate phase begins after both the source and release of radioactive effluents have been brought under control until additional protective measures are no longer needed. In the emergency phase, monitoring strategies aiming to identify people with high levels of exposure that need urgent decontamination and/or medical assessment and treatment should be developed. In the intermediate phase, the strategies to identify the priority population for further monitoring and possible treatment should be extended, which would inform the personal and public health counter-dose measures that aim to reduce exposure risks to radiation (e.g., shipping restrictions on agricultural products, radiation counseling, and dietary/lifestyle modification) [4,5,6].

The representativeness of the results of individual radiation monitoring is an important determinant of whether dose levels and other statistics estimated from such monitoring can be generalized to populations in affected areas [7]. In other words, representativeness can refer to the ability of the monitoring to accurately describe dose levels in the affected people over time, and their distribution in the population by place and person [8,9]. It would serve not only to adequately evaluate the effects and potential consequences of the radiological incident as well as the effectiveness of the counter-dose measures, but would also facilitate the production of quality standards and meaningful benchmarks for effective measures. Thus, representativeness needs to be achieved.

With regard to the Fukushima nuclear incident, it is important to note that voluntary participation, rather than mandatory, was adopted by the central and local authorities after the incident as the default scenario for individual-based radiation monitoring, in particular for adults [10]. However, the results of voluntary monitoring may pose a significant challenge of representativeness to the whole population of affected individuals because of voluntary response bias (namely, sampling bias) that often occurs when the sample consists of volunteers. Indeed, our previous report evaluating monitoring participation rates and behavior following the Fukushima incident has acknowledged that the participation rates differed significantly by age, sex, pre- and post-incident dwelling area, evacuation history, and air dose rate at the post-incident dwelling area [11]. These differences could be largely explained by different radiation risk perception and accessibility to the monitoring among the population [11], which may cause response bias to the monitoring, resulting in potentially limited application of the monitoring findings (dose levels and other statistics) to the whole population.

During the six-year-period following the Fukushima incident, the levels of external/internal exposure risk in the affected areas have been evaluated by many research organizations and are well documented. Except for dose reconstruction estimation with scientific assumptions [12,13], most of these evaluations were based on voluntary-based radiation monitoring administered by local authorities, using an individual radiation dosimeter for determining the external exposure level [3,14,15] or anthropogammametry (i.e., whole body counting (WBC) for determining the amount of radionuclides within the body at the time of monitoring) [16,17,18,19]. In summary, as a result of the counter-dose measures as well as natural removal phenomena such as weather and radioactive decay [6], the current exposure risks from radiation in the environment (attributable to the Fukushima incident) is considerably low or at undetectable levels [20], implying a marginal risk of radiation-associated physical health consequences of the incident [12,13]. However, to our knowledge, no rigorous studies have been performed to evaluate whether the results of these voluntary-based monitoring can be representative of the whole population in the affected areas.

Although a range of protective measures are still in place in some areas, it has been years since Japan entered the post-intermediate, recovery phase, which is the period that ends once the exposure risks to radiation have been confirmed to be reduced to acceptable, satisfactory levels for the public [4]. Currently, Japan is at a juncture towards the end of the recovery phase and beyond. We have to start thinking about whether to terminate the radiation monitoring or adopt the option that we proposed in our previous report [11], which is to shift the monitoring purpose from screening and dose assessment to concern/anxiety reduction over radiation contamination by utilizing the radiation monitoring as a risk communication tool to approach those who may be concerned about radiation risks [11,21]. Therefore, to support societal decision-making on the continued operation of the monitoring program under the current low exposure levels, rigorous evaluation of the results of the voluntary-based radiation monitoring, particularly with regard to their representativeness, is needed urgently.

Located 10–40 km (south to north) from the Fukushima nuclear power plant, Minamisoma City is unique in that it was the first city to have launched a voluntary-based internal radiation monitoring after the incident for its residents, which was done within four months following the incident (July 2011). With the cooperation of the Minamisoma City Office and the two WBC-equipped hospitals in the city, this study has two-fold objectives: To provide modeled estimates of the internal contamination levels in the whole population in the city over the 6 years after the Fukushima incident, based on the available data obtained from the voluntary participants of the monitoring (objective#1); and to assess the agreement between modeled (whole population) versus actually measured (voluntary participants) levels of internal contamination to examine if, and to what extent, the monitoring results from the voluntary participants can represent the whole population in the city (objective#2).

## 2. Materials and Methods

### 2.1. Setting 

**Figure 1:**
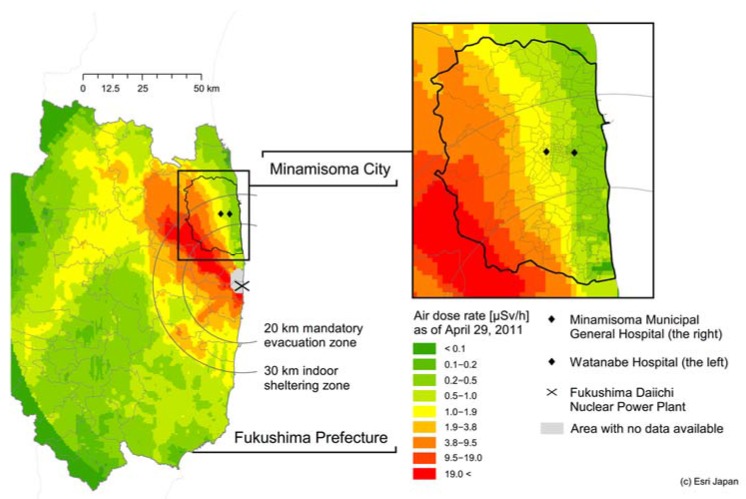
Location of Minamisoma City relative to the Fukushima Daiichi Nuclear Power Plant.

We have been supporting clinical care and research in Minamisoma City. On 12 March 2011, a mandatory evacuation instruction was issued within the 20 km radius of the Fukushima nuclear plant by the central government in alignment with the declaration of a nuclear emergency situation in response to the Fukushima nuclear incident [22]. On 15 March 2011, residents living within a 20–30 km radius from the plant were instructed to seek shelter indoors (i.e., indoor sheltering instruction) [22]. As a result of these initial protective measures, the post-incident decline of the population in Minamisoma City was substantial, reaching its lowest level on 22 March 2011, 11 days following the incident, to 7107 people, about 10% of the pre-incident population of 71,561 people (as of 9 March 2011) [23]. On 12 July 2016, the central government lifted the protective measures for all but a small portion of the city. The geographical scope of these evacuation and sheltering areas, and the locations of Minamisoma City and the two WBC-equipped hospitals, relative to the nuclear power plant, are shown in Figure 1.

### 2.2. Data Collection

Data were extracted from two databases administrated by Minamisoma City: Section 2.2.1. Whole Body Counter Database and Section 2.2.2. Evacuation Database, both described below.

#### 2.2.1. Whole Body Counter Database

After the Fukushima incident, Minamisoma City launched a voluntary-based internal radiation monitoring program for the city residents in July 2011, which was the initial monitoring initiative by the local authority in Japan post-incident. In the first three months of the monitoring program (from July 2011 until September 2011), two chair-type WBC units (Anzai Medical Co., Ltd., Tokyo, Japan and Fuji Electric Co., Ltd., Tokyo, Japan) were operated at the city hospital Minamisoma Municipal General Hospital (MMGH). In late September 2011, these WBC units were replaced by a standing-type WBC (FASTSCAN Model 2251, Canberra Inc., Meriden, CT, USA) due to their insufficient shielding against background gamma rays [18]. In July 2012, another chair-type WBC unit (WBC-R43-22458, Hitachi Aloka Medical, Ltd., Tokyo, Japan) was introduced at Watanabe Hospital, a private hospital in the city, which is located about 3 km to the west of MMGH. 

Monitoring is free of charge (except for travel expenses), by appointment only, and available to the general public aged 6 years or older because the premises of the WBC units were not optimal for obtaining measurements from small children. A program notification was sent to each household, including former residents who had evacuated elsewhere post-incident and could be tracked. The notification was also disseminated on the hospital website and the city’s public relations magazine. More details of the conduct of the WBC monitoring program in the city can be found elsewhere [20,24].

We obtained the data for each WBC participant from 1 October 2011 to 31 March 2015. We considered the WBC data exclusively from October 2011, measured by FASTSCAN at MMGH or WBC-R43-22458 at Watanabe Hospital. The data comprised of personal identification number, date of measurement, and results of the measurement (i.e., body burden (Bq/body) of radiocesium (Cs) 137 and 134). Note that radioactive Cs is one of the major constituents of an aerial release after a radiological incident, with long half-lives of 30 and 2 years for Cs-137 and Cs-134, respectively, and is known to be representative of total internal dose after a major nuclear incident [25]. The detection limits of the WBC units in both MMGH and Watanabe Hospital following a two-minute scan are 250 Bq/body for Cs-137 and 220 Bq/body for Cs-134. A team at the National Institute of Radiological Sciences investigated the WBC units using four sets of Bottle MAnnikin ABsorber (BOMAB) phantoms (Co-60, Cs-137, Ba-133, and water, manufactured by the Japan Radioisotope Association), and the overall efficiency of the detectors was accurate to within 10% [20].

#### 2.2.2. Evacuation Database

In this study, we defined the whole population of Minamisoma City as all individuals who were registered on the city’s resident register (described below) at a given time point. Modeling the internal contamination levels in the whole population of the city requires accurate data of post-incident population demographics (i.e., where a person lived and at what time point); this requirement is due to the area of residence could be an important indicator of internal contamination levels that should be considered in the estimation model (described below), although the levels of soil contamination alone were not considered to necessarily be correlated to levels of internal contamination after the Fukushima incident [26].

In Japan, there is the nationwide resident-registry network, knows as the Basic Resident Register, administrated by each municipality unit (city/town/village). This register contains basic data on registered residents, such as name, sex, date of birth, and address. Here, it is important to note that after the incident, because evacuees often did not change the address recorded in the Basic Resident Register of their original municipality, the registered residential address post-incident does not necessarily indicate that they actually lived at that address (referred to as “actual lived-at address”). However, evacuees did report their evacuation status, including actual lived-at address, to the Minamisoma City Office to receive important notifications from the office, such as tax payment, disaster recovery insurance, and compensation claims. Subsequently, in 2015, Minamisoma City created an Evacuation Database by combining such individuals’ evacuation records and their corresponding Basic Resident Register data. Although the evacuation-record reporting was not compulsory, given the legal necessity to receive tax and insurance notices, we considered that this database was complete, and contained the evacuation records of all Minamisoma residents. 

From this database, we could extract the following data for each individual in the whole population of Minamisoma City at eight time points (every 6 months) from 1 December 2011 to 1 June 2015: The personal identification number (to enable linkage to the WBC data, if participated in the monitoring), age (classified in 10-year intervals: 0–10, 11–20, 21–30, 31–40, 41–50, 51–60, 61–70, 71–80, and ≥81), sex, and actual lived-at address. In addition, to consider an original residential address in the city at the time of the incident (11 March 2011), we also obtained the registered residential address as of 1 March 2011, from the Basic Resident Register. Thereby, those who were not the city residents at the time of the incident were not considered in this study.

### 2.3. Eligible Age Groups

Since April 2013, compulsory participation in the WBC monitoring was required of all primary (ages 7–12 years) and secondary (13–15 years) school children in the city, in the form of an annual school health check-up, to respond to parents’ concerns over radiation in children. Because the scope of our study focused on the results of voluntary-based monitoring, we only considered those aged 21 years or older (i.e., age groups of 21–30 years to ≥81 years). Note that no direct comparison is possible between compulsory and voluntary participants in school children (i.e., no voluntary participants) and between compulsory participants in school children versus voluntary participants in other population (e.g., adults aged ≥21 years) because it is well known that children and adults have totally different risk of internal radiation exposure. The biological half-life of cesium (the time taken for half of a radioactive material to be eliminated from the body through biological processes), about 110 days for adults, is shorter in children [27].

### 2.4. Data Analysis

#### 2.4.1. Modeled Estimates of the Probability of Cs Detection in the Whole Population (Objective#1)

We performed three steps to estimate and evaluate the internal contamination levels in the whole population. Step 1: We modeled the individual-level relationships between the WBC participant’s data (independent variables) and their internal contamination levels (outcome variable), using multivariable regression techniques. Step 2: The whole population’s data were fitted to the models constructed in the step (1) to project the model-fitted internal contamination levels of each individual in the whole population. Step 3: The averages of the modeled internal contamination levels were calculated across the whole population to yield the population mean of the contamination levels.

Factors known or suspected of being associated with monitoring participation rates and participation behavior, as well as Cs detection, that were available in both the WBC participants and the whole population [11], were considered as independent variables in this study. They include age at a given time point (10-year intervals); sex (men and women); post-incident actual lived-at address at a given time point (categorized into four areas: Inside Minamisoma City, outside Minamisoma City but inside Fukushima Prefecture, neighboring prefectures of Fukushima, and outside the neighboring prefectures); and pre-incident residential addresses (formed into sub-district level). 

Note that post-incident actual lived-at addresses were de-identified prior to the data collection for participants to remain anonymous; thus, we were not able to access the full addresses. Instead, we obtained ones categorized into four areas as described above. Pre-incident residential addresses were also anonymized prior to the data collection by recoding into ones at the Oaza level (i.e., sub-district), which is the lowest hierarchy of administrative divisions of Minamisoma City. Note that Minamisoma City is structured into a hierarchy of administrative divisions: From the highest to the lowest: Ku (i.e., area, *n* = 3), Chiku (i.e., district, *n* = 12), and Oaza (i.e., sub-district, *n* > 100). It should be of note that our previous study demonstrated that the detection of Cs was not significantly associated with participation behavior (i.e., whether or not a resident would undergo a subsequent WBC monitoring) [11].

In addition, as an independent variable, we also took into account air dose rates (μSv/h), at a height of 1 m above ground measured in terms of the ambient dose equivalent (H*(10)) at pre-incident residential address [28], which were obtained from the official website of the Ministry of Education, Culture, Sports, Science, and Technology (MEXT) and pre-incident residential address, as an independent variable. Since the incident, the MEXT has irregularly performed airborne monitoring at 300 m above ground with a track width of about 1.85 km. All monitored results are accessible to the public and available online [29]. The dose rates were averaged by a 500 m^2^ mesh based on the Japan Profile for Geographical Information Standards (elevation and slope angle fourth mesh data) released by the Ministry of Land, Infrastructure and Transport [30]. Each WBC participant, therefore, belonged to one of the mesh areas. We considered the data of the first MEXT monitoring performed between 6 and 29 April 2011 (converted value as of 29 April 2011) [29].

We employed a dichotomous endpoint for internal contamination (i.e., detected or not detected for Cs-137 and Cs-134) as an outcome variable, rather than a continuous scale of body burden (Bq/body). The detection limit of Cs-137 and Cs-134 are 250 and 220 Bq/body following a 2-min scan, respectively. Note that because of the small number of independent variables that we could consider (see above) and large variances of the body burden (Bq/kg), we were unable to construct robust models that sufficiently explained the relationships between such continuous outcome measures (i.e., body burden (Bq/kg)) and independent variables. Therefore, logistic regression models were an appropriate analytical technique in the present study. This method allowed us to project the probability of Cs detection, based on the values of the independent variables, which was then the outcome measure of interest in this study. Pre-incident residential address was used as a random effect (as long as stable models were obtained) to take into account geographical similarities or differences in the outcome for the regressions.

The modeled probability of Cs detection covered the following 11 time periods: October 2011; November 2011; December 2011; January 2012; February 2012; March to April 2012; May 2012; June 2012; July to August 2012; September 2012 to March 2013; and April 2013 to March 2015. These sets of time periods were considered to have a sufficient number of Cs-detected individuals (approximately 100) in each period, so that stable models were obtained. Note that because the relationships (magnitude and significance) between internal contamination and independent variables are considered to be not consistent during the study periods [11], the finely-divided study periods were preferable, which is also clinically meaningful. Models were constructed separately for Cs-137 and Cs-134 for these time periods. For independent variables (e.g., age and post-incident actual lived-at address) in models at each time period, data from the closest time point recorded in the Evacuation Database (every 6 months from 1 December 2011 to 1 June 2015) were used.

#### 2.4.2. Robustness of the Regression Models

Robustness of the regression models were examined by fitting the WBC participants’ data to the models constructed in the step (1) to project the model-fitted probability of Cs detection of each WBC participant, which allowed for the assessment of the models’ prediction accuracy in terms of agreement between the modeled probability of Cs detection and actually measured Cs detection rates among the WBC participants. The mean of modeled estimates of the detection probability in the WBC participants was compared with the actual detection rates among them, using the *t*-test. Comparisons were performed separately for Cs-137 and CS-134, and by sex and time point (as defined above).

#### 2.4.3. Comparison between Population Mean of Modeled Probability of Cs Detection versus Actual Detection Rates in the WBC Participants (Objective#2)

Finally, to examine if and the extent to which the monitoring results from the voluntary participants can represent the whole population in the city, we assessed the agreement between modeled probability of Cs detection in the whole population versus actual Cs detection rates in the WBC participants. Comparisons were conducted in the same manner as the above-mentioned assessment of robustness of the regression models.

#### 2.4.4. Sub-Data Analysis 

Because our study was based entirely on the available data obtained from the participants of the WBC monitoring, we were unable to consider in the regression models potential differences in the internal contamination levels between the WBC participants and those who did not participate in the WBC monitoring (non-participants). In other words, we had no alternative in this study but to assume equal distribution of internal contamination levels between the WBC participants and non-participants.

Two factors could have potentially violated this assumption: (1) WBC participants were more concerned about radiation with reason (i.e., participated in WBC monitoring because they had higher levels of internal contamination); and (2) WBC participants were more concerned about the effects of radiation, and were more likely to adopt personal counter-dose measures (e.g., dietary/lifestyle modification, such as consumption of homegrown foods with radiation inspection) and thus, had lower levels of internal contamination. However, it was unknown which direction exerted a stronger influence (bias) on the monitoring results, or rather, they might have been offset by each other.

Although it was impossible to directly verify the effect of the above factors by comparing the internal contamination levels between the WBC participants and non-participants, we adopted the following two alternative approaches: (a) comparison of the probability of Cs detection after April 2013 (1 April 2013 to 31 March 2015) between those who participated and those who did not participate in the WBC monitoring before April 2013 (1 September 2011 to 31 March 2013); and (b) vice versa, i.e., the comparison of the probability of Cs detection before April 2013 between the WBC participants and non-participants after April 2013. Note that when the WBC monitoring started in July 2011, appointments were booked more than a half year in advance. Because of the large number of pre-booked appointments, residents could not usually participate in the monitoring more than once until March 2013. Thereafter, since April 2013, as waiting times for appointments decreased slightly, it was possible for participants to have repeat measurements in the same year. 

For this comparison, as independent variables, we also collected data on weight and height at the time of WBC monitoring, which are only available in the Whole Body Counter Database. Multivariable logistic regression models were constructed (for Cs-137 and Cs-134, separately) to examine the potential differences in odds for Cs detection before/after April 2013 between those who participated in the WBC monitoring and those who did not before/after April 2013, after adjusting for covariates (independent variables), such as age, sex, weight, height, post-incident actual lived-at, pre-incident residential address, and air dose rate as of 29 April 2011, at a pre-incident residential address. As some individuals participated in WBC monitoring more than once after/before April 2013, the first monitoring data were considered. Post-incident actual lived-at address was also considered as a random effect in the models.

We used STATA/MP version 14.2 (StataCorp LLC, College Station, TX, USA) for all analyses, and a *p*-value of less than 0.05 was considered statistically significant.

### 2.5. Ethics Approval

Ethical approval for this study was granted by the ethics committee of the Minamisoma Municipal General Hospital in Minamisoma City; approval number 28-02. The ethics committee agreed that written consent was not required for each individual because this study was performed retrospectively.

## 3. Results

### 3.1. Demographic Characteristics

**Figure 2:**
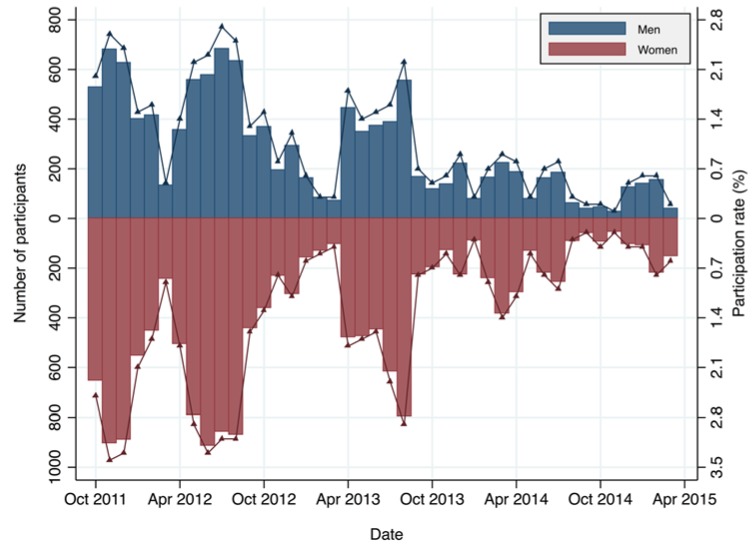
Monthly number of while body counter monitoring participants aged 21 years and older and participation rates (%) in the whole population by sex. Bar: Number of participants (left Y-axis), solid line: Participation rate (right Y-axis).

During the study period from 1 October 2011 to 31 March 2015, there were a total of 26,992 participation (19,263 individuals) in the WBC monitoring (men: 42.9%, women: 57.1%). The age group of 61–70 years accounted for the largest proportion of the participants (25.1%), followed by 51–60 years (19.7%), and 71–80 years (15.1%). The distribution of WBC participants by age group and sex is shown in Table 1, and Figure 2 illustrates the distribution of the monthly number of WBC participants by sex. Several peaks, for example, at December 2011, June 2012, August 2013, were seen that were due to the effects of the program notifications issued around these times by the Minamisoma City Office.

From the Evacuation Database, we determined the whole population (aged 21 years and older) to be 53,982 people (women: 52.1%) from October 2011 to May 2012. Among this population, arithmetic mean air dose rate as of 29 April 2011 at pre-incident residential address was 1.2 (μSv/h) with standard deviation of 1.5. The whole population was also determined to be 53,096 people (women: 52.1%) from June to November 2012; 52,723 people (women: 52.1%) from December 2012 to May 2013; 51,971 people (women: 52.2%) from June to November 2013; 51,541 people (women: 52.1%) from December 2013 to May 2014; 50,975 people (women: 52.2%) from June to November 2014; and 50,575 people (women: 52.2%) from December 2014 to March 2015. Figure 2 also shows the monthly participation rates of the WBC monitoring per population, ranging from 2.7% in July 2012 (highest) to 0.1% in November 2014 (lowest) in men, and from 3.4% in November 2011 (highest) to 0.2% in November 2014 (lowest) in women.

**Figure 3:**
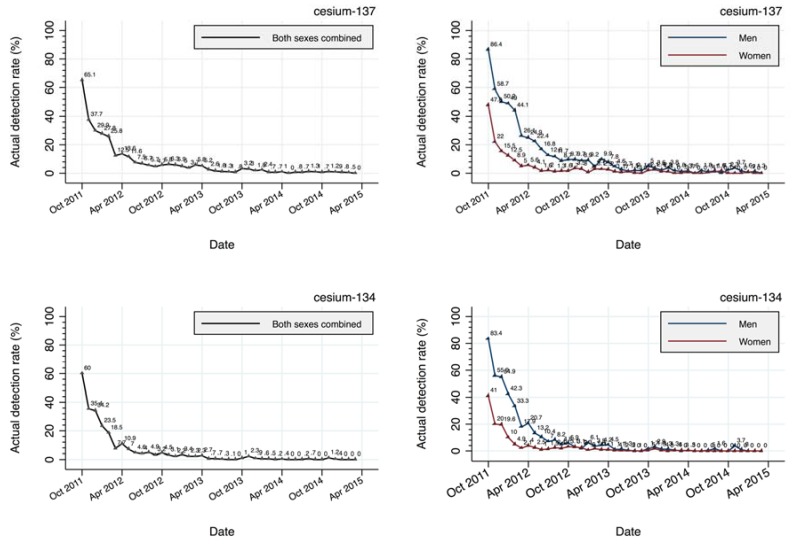
Monthly actual detection rates in both sexes combined (**left**) and in men and women (**right**) for Cs-137 (**upper**) and Cs-134 (**lower**) in the WBC participants.

Figure 3 illustrates the actual monthly detection rates of Cs-137 and Cs-134 during the study period. In October 2011, 6 months following the Fukushima incident, the detection rates of Cs-137 and Cs-134 were 65.1% (men: 86.4%, women: 47.8%) and 60.0% (men: 83.4%, women: 41.0%), respectively, which declined dramatically in a half year to approximately 10% for both radionuclides, by around March 2012. Thereafter, the rate of the decline had stagnated, and the detection rates were around 1% or less since April 2013, and close to zero since April 2014, 6 years after the incident. Over the years, men had substantially higher detection rates than women, particularly in the first 2 years following the incident.

### 3.2. Regression Models to Project the Probability of Cs Detection in the Whole Population

Regression models developed using the data of the WBC participants were shown in Supplementary Table S1, which were used to project the probability of Cs detection for each individual in the whole population. Models were constructed separately for the different time periods and for Cs-137 and Cs-134. In summary, estimated relationships between independent variables and Cs detection have been mostly consistent over the study period. Higher probabilities of Cs detection were observed in the elderly than in young adults, men than in women, and those who lived inside Minamisoma City after the incident than those living in or outside the neighboring prefectures of Fukushima post-incident. Higher air dose rate at pre-incident residential address was also associated with higher probability of Cs detection. Odds for Cs detection among the elderly against young adults showed a gradual increase over the years, indicating that age gaps in risks from and control measures against internal contamination has increased with time.

### 3.3. Robustness of the Regression Model

Any *t*-test comparisons (i.e., separately for Cs-137 and Cs-134, and by sex, and time points) between the mean of modeled Cs detection probability in WBC participants (estimated by fitting their data to the constructed models) and their actual detection rates demonstrated no statistical significance, indicating robustness of the models (data not shown).

### 3.4. Modeled Estimates of the Probability of Cs Detection in the Whole Population (Objective#1)

**Figure 4:**
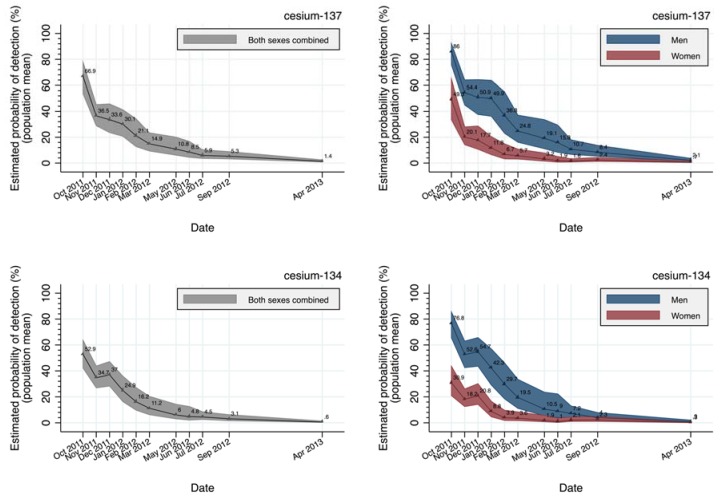
A population mean of modeled estimates of the probability of Cs detection and the corresponding 95% confidence band in both sexes combined (**left**) and in men and women (**right**) for Cs-137 (**upper**) and Cs-134 (**lower**). The confidence band was demarcated by the population mean of the upper and lower limit of the confidence intervals of modeled probability of Cs detection. The point at April 2013 covers the time period until March 2015.

The population mean of the modeled estimates of the probability of Cs detection were plotted over time (Figure 4). For both sexes combined, the population mean in October 2011 was 67.0% and 53.0% for Cs-137 and Cs-134, respectively; the population mean for its 95% confidence intervals (CI) were 53.8–78.1 and 42.3–63.9, respectively. As with the actual detection rates, a substantial sex difference in Cs detection probability in the population mean was observed in October 2011: For Cs-137, men: 86.0% (75.6–92.3) and women: 49.3% (33.5–65.0); for Cs-134, men: 76.8% (65.4–85.6) and women: 30.9% (20.9–43.7). The rate of decline of Cs detection probability had stagnated since around March 2012, and the probability was about 1% or less after April 2013.

### 3.5. Agreement between Modeled (Whole Population) versus Actually Measured (Voluntary Participants) Probability of Cs Detection (Objective#2)

Table 2 shows *t*-test comparisons between population means of modeled estimates of the probability of Cs detection and actual detection rates in WBC participants. 

There was better representativeness of the WBC participants’ data, regarding the agreement between the modeled probabilities (in whole population) versus actual rates (in WBC participants) of Cs detection, in women than in men. In women, no significant differences in the comparisons were observed for Cs-137, while the mean of the modeled estimates were significantly lower than actual detection rates by 10.1% (95% CI 6.3–13.9; *p* < 0.001) for Cs-134 in October 2011. In men, likewise, significantly lower levels of Cs-134 detection were demonstrated in the mean modeled probabilities than in actual rates in October 2011 by 6.5% (95% CI 3.3–9.8; *p* < 0.001). In addition, we found lower levels of Cs detection in the mean modeled probability than in the actual rates at some time periods for both Cs-137 and Cs-134; although the differences were small. 

### 3.6. Sub-Data Analysis

Table 3 shows the regression models that examined the potential difference in the probability of Cs detection after April 2013 between those who participated in the WBC monitoring and those who did not before April 2013. After adjusting for covariates, no statistically significant differences in the odds ratios were identified between Cs-137 and Cs-134, although larger odds for Cs detection was estimated in the non-participants than in the participants (Cs-137: 1.28 (95% CI 0.92–1.77); Cs-134: 1.69 (95% CI 1.00–2.84)). Similarly, the odds ratios for detection of Cs-137 and Cs-134 before April 2013 in the non-participants after April 2013 in relation to the participants were 1.02 (95% CI 0.92–1.13) and 1.11 (95% CI 1.00–1.23), respectively, with no statistically significant differences (Table 4). These results might support evidence of equal or close distributions of internal contamination levels between the WBC participants and non-participants.

## 4. Discussion

We estimated the probability of Cs detection in the whole population in Minamisoma City over the six years following the Fukushima nuclear incident, and assessed the agreement between the modeled estimates of the probability of Cs detection and the actually measured Cs detection rates among the participants in voluntary WBC monitoring. This allowed for investigating the extent to which the data of voluntary participants in WBC monitoring could represent the whole population. Of approximately 70,000 individuals (60,000 aged ≥21 years) residing in Minamisoma City before the incident, a total of 19,263 residents participated in the WBC monitoring (26,992 participation) during the study period from 1 October 2011 to 31 March 2015. In October 2011, the population means of the modeled estimates of the probability of Cs detection were 67.0% (men: 86.0%, women: 49.3%) and 53.0% (men: 76.8%, women: 30.9%) for Cs-137 and Cs-134, respectively, which declined dramatically within 1 year following the incident. 

Given the physical half-life of Cs-137 and Cs-134 (30 and 2 years, respectively), the internal contamination in the first year of the incident could be largely attributable to the acute intake of radioactive Cs immediately after the incident as well as radionuclide inhalation, while that in the following years could be largely attributed to the chronic ingestion of Cs contaminated food [31]. As shown in Supplementary Table 1 (i.e., the modeled relationships of Cs detection in WBC participants to their demographics, which was used to project the probability of Cs detection in the whole population), we found that higher Cs detection rates have been identified in the elderly individuals than in young adults. One possible reason is that given that kidneys are the main organ of exertion of Cs out of the organism, it is possible to confirm that the elderly people have a lower excretory function than young adults, which may lead to the accumulation of Cs in their organism. Men also had higher Cs detection rates than women. It is known that women have higher health risk perception than men [32], which could possibly explain why those who have a higher radiation risk perception are more likely to refrain from consuming potentially highly contaminated unmonitored foods (i.e., no radiation inspection), resulting in lower internal contamination levels. Cs detection rates were also associated with post-incident actual lived-at address with those who lived in or outside the neighboring prefectures of Fukushima post-incident were more likely to have lower detection rates than those living within the city. Higher air dose rate at a pre-incident residential address was also associated with a higher probability of Cs detection. These findings may indicate that those living in contaminated areas were at a relatively high risk of being exposed to the chance of consuming potentially contaminated unmonitored foods than those living in less contaminated areas. Note that just the levels of soil contamination—A major indicator of air dose rate—Were acknowledged as not necessarily contributable to levels of internal contamination after the Fukushima incident [26], while individual-level dietary and lifestyle habits/preferences had greater impacts on the contamination levels [26].

As shown in Figure 4 (i.e., modeled estimates of the probability of Cs detection), the rate of decline in the estimated probability of Cs detection has stagnated since around April 2012, and the estimated probability was about 1% or less after around April 2013. Table 2 (i.e., comparison between modeled (whole population) versus actually measured (voluntary participants) probability of Cs detection) also demonstrated that the population means of the modeled estimates of probability of Cs-134 detection were significantly lower than actual detection rates by 7.1% for both sexes combined (men: 6.5%, women: 10.1%) in October 2011, while Cs-137 showed no statistically significant difference between them for both sexes. For both Cs-137 and Cs-134, other subsequent time periods showed sufficient agreement between the modeled probabilities versus actual Cs detection rates, indicating good representativeness of the voluntary WBC participant group. Although disagreement was observed between the groups for men in some time periods, with borderline significance.

### 4.1. Voluntary-Based Participation: Very Real Possible Default Setting for Individual Radiation Monitoring after a Nuclear Incident

After a major nuclear incident, the default settings for the individual radiation monitoring (i.e., whether monitoring participation should be mandatory or voluntary) is pivotal in designing a monitoring strategy. The choice of the default setting can lead (i.e., nudge [33]) people to create/increase or moderate their anxieties and concerns over radiation exposure [10], determining the overall public health harms/benefits of monitoring [10]. At the emergency phase of the Fukushima incident, there were many unknowns, not only about the practical and technical aspects of the monitoring, but also the future exposure risks in terms of likelihood, severity, health impact, and more. Given that context, voluntary participation was adopted by the central and local authorities as the default setting for the monitoring, particularly of the adults [10,21], which reconciled with libertarian paternalism [33,34], a concept derived from cognitive psychology and behavioral science, which aims to encourage individuals to make choices that are more in line with their long-term best interests, while maintaining their freedom of choice. Libertarian paternalism is probably the phrase that best describes the approach that the bureaucratic system of many Western countries (including the United Kingdom and the United States of America), as well as Japan, are likely to adopt soon for its citizens [10,35]. This means that there is a very real possibility that future nuclear incidents in the world will adopt the same strategy as Japan had done in response to the Fukushima incident, with respect to adopting voluntary individual radiation monitoring, rather than mandatory, for the establishment of a resilient radiation protection and safety program. In other words, in future nuclear incidents, mandatory radiation monitoring that would have high/complete participation rates with low risk of bias and confounding is not realistic approach to assess dose levels in the affected population. So, a novel approach should probably be considered which can project the whole population based on very limited voluntary samples. In this context, the lessons learnt from the Fukushima incident about whether the monitoring results from the voluntary participants can represent the whole population will be invaluable and exert a powerful, informative influence on the management of such an incident in future.

### 4.2. Good Representativeness of the Voluntary Radiation Monitoring—Clinically Meaningful as a Screening and Dose Assessment Tool

One important implication of our results is that, except in October 2011 (6 months following the incident, and the inception of the WBC monitoring in the city), sufficient agreement between modeled (whole population) versus actually measured (voluntary participants) Cs detection rates were observed for both sexes throughout the study periods (Table 2), indicating good representativeness of the voluntary participants in WBC monitoring. Thus, voluntary internal monitoring is clinically meaningful as a screening and dose assessment tool.

The disagreement for Cs-134 in October 2011 might be largely due to different distributions of the demographic characteristics between the WBC participants and non-participants, namely, demographic gaps. Our previous study acknowledged that participation behavior for the voluntary monitoring was significantly associated with age, sex, pre- and post-incident dwelling area, evacuation history, and air dose rate at the post-incident dwelling area, while detection of Cs was not significantly associated with monitoring participation behavior [11]. For example, in comparison with the age cohort of 21–30 years, the cohort of 71–80 and ≥81 years were less likely to participate in the monitoring; women had higher participation rates than men; and those who were living in evacuation zones at the time of the incident had higher monitoring participation rates than those who lived outside any of the evacuation zones [11]. Therefore, because the modeled estimates of the Cs detection probability were computed based only on the relationships of Cs detection in WBC participants to their demographics, these demographic gaps between WBC participants and non-participants might result in such disagreement between modeled probabilities and the actual detection rates (Table 2). In addition, Cs detection rates in WBC participants were relatively higher at this time (compared to other subsequent time periods), which might make it easier to detect the disagreement with statistical significance. Note that although the demographic gaps between the WBC participants and non-participants have existed over the 6 years (data not shown), because the Cs detection rates in the WBC participants were relatively low in these periods (compared to October 2011), the gaps did not cause much disagreement between modeled probabilities and the actual Cs detection rates, which also made it difficult to detect statistical significance. It was unclear why only Cs-134 had a significant disagreement in October 2011, while no significant disagreement was found for Cs-137. One possible reason may be that owing to the large difference in the physical half-lives of Cs-137 and Cs-134 (30 and 2 years, respectively), their distribution patterns and variations within the environment might differ substantially. This might make the effects of the demographic gap (particularly in post-incident actual lived-at address) between the WBC participants and non-participants on the agreement between the modeled probabilities and actual detection rates also different between Cs-137 and Cs-134, in particular, at the time when detection rates were high.

### 4.3. Towards a Long-Term Strategy for Voluntary Radiation Monitoring, and Potential as a Risk Communication Tool for Mental Health

Other considerations include the estimated probability of Cs detection in the whole population, which has been around only 1% or less since April 2013, 2 years following the incident. Here, we would like to echo the points proposed in our previous study that the radiation monitoring can include or shift its purpose from screening and dose assessment to concern/anxiety reduction over radiation contamination by utilizing the radiation monitoring as a risk communication tool to approach those who may be concerned about radiation risks [11,21]. In contrast with the findings of only marginal internal radiation contamination in the public, it appears that the increasing burden of mental health problems may outweigh the radiation risks [35,36]. Anxiety about radiation risks can cause psychological distress [37], in particular, among forced-evacuees post-nuclear incidents [38]. In this regard, Minamisoma City is a good case example, in that monitoring participants in the city with Cs-137 contamination of more than 50 Bq/kg were offered counseling from hospital physicians about risky food intake and advised to refrain from consuming potentially highly contaminated unmonitored foods [39]. Our previous study that evaluated the factors associated with the monitoring participation behavior should help enhance the discussion around the types of individuals that the internal radiation monitoring can or should approach to use as a risk communication tool to alleviate anxiety and support risk reduction measures [11]. 

### 4.4. Limitations

Our analyses were subject to some limitations. First, our regression projection/estimates for the whole population was based only on the data from the WBC participants, which means that our findings were derived under the assumption that internal contamination levels were equally distributed between the WBC participants and non-participants. In this regard, our sub-data analysis verified no statistical significance in the difference in odds for Cs detection after April 2013 between those who participated in the WBC monitoring and those who did not before April 2013, and vice-versa also showed no statistical significant differences (Table 3 and Table 4). However, this verification was not able to consider those who did not attend the WBC monitoring throughout the study periods; hence, our assumption was not fully confirmed. Second, ideally, the evaluation of the internal contamination should be communicated in the context of the body burden (Bq/body) or dose, which provides more informative inputs for risk management of internal exposure, rather than the dichotomous endpoint (i.e., Cs detection). However, as we greatly lacked variables available to be incorporated into the regression models, we were unable to construct models that sufficiently explained the large variance in continuous scale of body burden (Bq/body). By implementing a survey that can extract additional data (e.g., dietary and lifestyle habits/preferences known to be associated with internal contamination level [39]) from the whole or randomly sampled population, including both the WBC participants and non-participants, robustness in building a model to explain Cs body burden, as well as detection probability, will be substantially improved. Note that given that dietary and lifestyle habits/preferences can be highly correlated to socio-demographic characteristics, such as age and sex (which are fully considered in this study), it is quite likely that our regression models largely explained the variations of Cs detection rates in the WBC participants; therefore, lack of these variables would not much affect our findings. Finally, because our study scope encompassed only Minamisoma City, we have limited ability to generalize to the wider population beyond the city. However, given that radiation-release incidents occur very rarely and only in very specific locations, the lessons learned about the conduct of internal radiation monitoring in Minamisoma City—The city that was heavily affected by the radiation plume and led the vanguard among the affected municipalities by launching the first voluntary internal monitoring program for a population in the city—Will be invaluable in managing such an incident better in future.

## 5. Conclusions

To our knowledge, this is the first study to evaluate the representativeness of voluntary internal radiation monitoring after Japan’s 2011 Fukushima nuclear incident over the six years post-incident. We found good representativeness of the voluntary monitoring group, affirming the clinical importance of the voluntary-based monitoring as a screening and dose assessment tool post-nuclear incident. Our intention in the present study was not to question the default setting of the monitoring (mandatory or voluntary), but to support societal decision-making regarding the long-term maintenance of the monitoring program under the current low exposure levels, and to inform the global planning and preparedness of the internal contamination monitoring programs for similar nuclear incidents in the future.

## Figures and Tables

**Table 1:** Distribution of whole body counter monitoring participants by age group and sex from 1 October 2011 to 31 March 2015.

Age Group (Year)	Men (% in Row)	Women (% in Row)	Total (% in Column)
21–30	791 (38.2)	1279 (61.8)	2070 (7.7)
31–40	1631 (41.6)	2285 (58.4)	3916 (14.5)
41–50	1586 (41.7)	2215 (58.3)	3801 (14.1)
51–60	2132 (40.2)	3172 (59.8)	5304 (19.7)
61–70	3062 (45.2)	3718 (54.8)	6780 (25.1)
71–80	1918 (47.0)	2163 (53.0)	4081 (15.1)
81–	473 (45.5)	567 (54.5)	1040 (3.9)
**Total**	**11,593 (42.9)**	**15,399 (57.1)**	**26,992 (100.0)**

**Table 2:** Comparison between population means of modeled estimates of probability of Cs detection and actual detection rate in whole body counter (WBC) monitoring participants, for both sexes combined (A), men (B), and women (C), for Cs-137 (the upper) and Cs-134 (the lower).

(A) Both Sexes Combined				
Cs-137				
	WBC Participants	Whole Population		
Time Period	Actual Detection Rate (%)	Population Mean of Modeled probability of detection (%) (95% CI) *	Difference (%) (95% CI) **	*p*-Value
October 2011	65.1	66.9 (66.7 to 67.0)	−1.8 (−4.6 to 0.9)	0.19
November 2011	37.7	36.5 (36.4 to 36.7)	1.2 (−1.2 to 3.6)	0.33
December 2011	29.9	33.6 (33.5 to 33.8)	−3.7 (−6.1 to −1.4)	<0.01
January 2012	27.8	30.1 (29.9 to 30.2)	−2.2 (−5.1 to 0.7)	0.14
February 2012	25.8	21.1 (21.0 to 21.3)	4.6 (1.7 to 7.6)	<0.01
March 2012 to April 2012	13.2	14.9 (14.7 to 15.0)	−1.6 (−3.5 to 0.3)	0.12
May 2012	11.6	10.8 (10.7 to 10.9)	0.8 (−0.9 to 2.6)	0.34
June 2012	7.5	8.5 (8.5 to 8.6)	−1.0 (−2.4 to 0.3)	0.16
July 2012 to August 2012	6.2	5.9 (5.9 to 6.0)	0.2 (−0.6 to 1.1)	0.60
September 2012 to March 2013	5.4	5.3 (5.2 to 5.3)	0.1 (−0.7 to 0.9)	0.78
April 2013 to March 2015	1.6	1.4 (1.4 to 1.4)	0.2 (0.0 to 0.5)	0.05
**Cs-134**				
	**WBC Participants**	**Whole Population**		
**Time Period**	**Actual Detection Rate (%)**	Population Mean of Modeled Probability of Detection (%) (95% CI) *	Difference (%) (95% CI) **	***p*-Value**
October 2011	60.0	52.9 (52.7 to 53.1)	7.1 (4.3 to 9.9)	<0.001
November 2011	35.4	34.7 (34.5 to 34.9)	0.7 (−1.7 to 3.1)	0.57
December 2011	34.2	37.0 (36.9 to 37.2)	−2.8 (−5.3 to −0.4)	<0.05
January 2012	23.5	24.9 (24.7 to 25.0)	−1.3 (−4.1 to 1.4)	0.34
February 2012	18.5	16.2 (16.1 to 16.4)	2.2 (−0.4 to 4.8)	0.08
March 2012 to April 2012	9.9	11.2 (11.1 to 11.3)	−1.3 (−3.0 to 0.4)	0.15
May 2012	7.0	6.0 (5.9 to 6.0)	1.0 (−0.4 to 2.4)	0.13
June 2012	4.6	4.8 (4.8 to 4.9)	−0.2 (−1.3 to 0.9)	0.73
July 2012 to August 2012	4.4	4.5 (4.5 to 4.6)	−0.1 (−0.9 to 0.6)	0.75
September 2012 to March 2013	3.2	3.1 (3.1 to 3.1)	0.1 (−0.5 to 0.8)	0.69
April 2013 to March 2015	0.6	0.6 (0.6 to 0.6)	0.0 (−0.1 to 0.2)	0.62
**(B) Men**				
**Cs-137**				
	**WBC Participants**	**Whole Population**		
**Time Period**	**Actual Detection Rate (%)**	Population Mean of Modeled Probability of Detection (%) (95% CI) *	Difference (%) (95% CI) **	***p*-Value**
October 2011	86.4	86.0 (85.9 to 86.1)	0.4 (−2.6 to 3.4)	0.79
November 2011	58.7	54.4 (54.2 to 54.6)	4.3 (0.5 to 8.0)	<0.05
December 2011	50.2	50.9 (50.7 to 51.1)	−0.6 (−4.6 to 3.3)	0.75
January 2012	49.0	49.9 (49.8 to 50.1)	−0.9 (−5.9 to 4.0)	0.71
February 2012	44.1	36.8 (36.7 to 37.0)	7.3 (2.4 to 12.1)	<0.01
March 2012 to April 2012	25.2	24.8 (24.7 to 25.0)	0.4 (−3.5 to 4.2)	0.85
May 2012	22.4	19.1 (19.0 to 19.2)	3.3 (−0.2 to 6.7)	0.05
June 2012	16.8	15.8 (15.6 to 15.9)	1.0 (−2.1 to 4.1)	0.51
July 2012 to August 2012	12.1	10.7 (10.6 to 10.8)	1.4 (−0.4 to 3.3)	0.10
September 2012 to March 2013	9.0	8.4 (8.3 to 8.5)	0.6 (−0.9 to 2.1)	0.41
April 2013 to March 2015	2.7	2.1 (2.0 to 2.1)	0.6 (0.1 to 1.2)	<0.01
**Cs-134**				
	**WBC Participants**	**Whole population**		
**Time Period**	**Actual Detection Rate (%)**	Population Mean of Modeled Probability of Detection (%) (95% CI) *	Difference (%) (95% CI) **	***p*-Value**
October 2011	83.4	76.8 (76.7 to 76.9)	6.5 (3.3 to 9.8)	<0.001
November 2011	55.9	52.6 (52.5 to 52.8)	3.2 (−0.5 to 7.0)	0.10
December 2011	54.9	54.7 (54.5 to 54.9)	0.2 (−3.8 to 4.1)	0.94
January 2012	42.3	42.3 (42.1 to 42.5)	−0.1 (−4.9 to 4.8)	0.98
February 2012	33.3	29.7 (29.5 to 29.8)	3.6 (−1.0 to 8.1)	0.12
March 2012 to April 2012	19.9	19.5 (19.4 to 19.6)	0.4 (−3.1 to 4.0)	0.81
May 2012	13.2	10.5 (10.4 to 10.6)	2.8 (−0.1 to 5.6)	<0.05
June 2012	10.4	9.0 (8.9 to 9.0)	1.4 (−1.1 to 3.9)	0.24
July 2012 to August 2012	7.6	7.2 (7.1 to 7.2)	0.4 (−1.0 to 1.9)	0.55
September 2012 to March 2013	4.4	4.0 (3.9 to 4.0)	0.4 (−0.7 to 1.5)	0.44
April 2013 to March 2015	1.0	0.9 (0.9 to 0.9)	0.2 (−0.1 to 0.5)	0.23
**(C) Women**				
**Cs-137**				
	**WBC Participants**	**Whole Population**		
**Time Period**	**Actual Detection Rate (%)**	Population Mean of Modeled Probability of Detection (%) (95% CI) *	Difference (%) (95% CI) **	***p*-Value**
October 2011	47.8	49.3 (49.1 to 49.4)	−1.5 (−5.4 to 2.4)	0.45
November 2011	22.0	20.1 (20.0 to 20.2)	1.8 (−0.9 to 4.6)	0.18
December 2011	15.5	17.7 (17.6 to 17.8)	−2.2 (−4.6 to 0.2)	0.09
January 2012	12.5	11.8 (11.7 to 11.9)	0.7 (−2.0 to 3.5)	0.59
February 2012	8.9	6.7 (6.6 to 6.7)	2.2 (−0.4 to 4.8)	0.06
March 2012 to April 2012	5.4	5.7 (5.6 to 5.7)	−0.3 (−1.9 to 1.3)	0.72
May 2012	4.1	3.2 (3.2 to 3.2)	0.8 (−0.6 to 2.2)	0.19
June 2012	1.6	1.9 (1.9 to 1.9)	−0.2 (−1.1 to 0.6)	0.59
July 2012 to August 2012	1.6	1.6 (1.6 to 1.6)	0.0 (−0.6 to 0.6)	0.91
September 2012 to March 2013	2.2	2.4 (2.4 to 2.4)	−0.2 (−0.9 to 0.5)	0.64
April 2013 to March 2015	0.8	0.7 (0.7 to 0.7)	0.1 (−0.2 to 0.3)	0.58
**Cs-134**				
	**WBC Participants**	**Whole Population**		
**Time period**	**Actual Detection Rate (%)**	Population Mean of Modeled Probability of Detection (%) (95% CI) *	Difference (%) (95% CI) **	***p*-Value**
October 2011	41.0	30.9 (30.8 to 31.0)	10.1 (6.3 to 13.9)	<0.001
November 2011	20.0	18.2 (18.1 to 18.3)	1.8 (−0.9 to 4.4)	0.18
December 2011	19.6	20.8 (20.7 to 20.9)	−1.2 (−3.8 to 1.5)	0.39
January 2012	10.0	8.8 (8.8 to 8.9)	1.1 (−1.4 to 3.6)	0.36
February 2012	4.9	3.9 (3.8 to 3.9)	1.0 (−1.0 to 3.0)	0.27
March 2012 to April 2012	3.4	3.6 (3.6 to 3.7)	−0.3 (−1.6 to 1.0)	0.68
May 2012	2.5	1.9 (1.9 to 1.9)	0.7 (−0.4 to 1.8)	0.18
June 2012	1.0	1.0 (1.0 to 1.0)	0.0 (−0.7 to 0.6)	0.97
July 2012 to August 2012	2.0	2.1 (2.1 to 2.1)	−0.1 (−0.8 to 0.5)	0.70
September 2012 to March 2013	2.2	2.3 (2.3 to 2.3)	−0.1 (−0.8 to 0.6)	0.83
April 2013 to March 2015	0.3	0.3 (0.3 to 0.3)	0.0 (−0.2 to 0.1)	0.87

Note: CI = confidence interval; * confidence intervals for the population mean of modeled probability; ** confidence intervals for the difference between the population mean of modeled probability and actual detection rate.

**Table 3:** Regression models to examine the potential differences in odds for detection of Cs-137 and Cs-134 after April 2013 between those who participated in the WBC monitoring and those who did not before April.

Variables	Cs-137 after April 2013	Cs-134 after April 2013
Whole body counter (WBC) monitoring participation before April 2013		
No	1.28 (0.92–1.77)	1.69 (1.00–2.84)
Yes	1.00	1.00
Age group (year)		
21–30	0.21 (0.05–0.89) *	NA
31–40	0.30 (0.12–0.76) *	0.32 (0.07–1.39)
41–50	0.36 (0.16–0.81) *	0.14 (0.02–1.02)
51–60	0.42 (0.23–0.77) **	0.23 (0.07–0.77) *
61–70	1.00	1.00
71–80	1.49 (1.01–2.21) *	1.37 (0.76–2.47)
81–	0.93 (0.46–1.87)	0.68 (0.22–2.08)
Sex		
Men	1.00	1.00
Women	0.25 (0.15–0.42) ***	0.22 (0.10–0.50) ***
Height at WBC measurement	0.98 (0.95–1.01)	0.96 (0.91–1.01)
Weight at WBC measurement	1.01 (0.99–1.02)	1.02 (0.99–1.05)
Post-incident actual lived-at address		
Inside Minamisoma City	1.00	1.00
Outside Minamisoma City but inside Fukushima Prefecture	0.60 (0.28–1.32)	NA
Neighboring prefectures of Fukushima	0.19 (0.03–1.36)	NA
Outside the neighboring prefectures	NA	NA
Air dose rate (μSv/h) as of 29 April 2011 at pre-incident residential address	1.23 (1.13–1.33) ***	1.23 (1.11–1.36) ***

Note: * *p* < 0.05; ** *p* < 0.01; *** *p* < 0.001. Variables in the tables were mutually adjusted. Pre-incident residential address was also adjusted as a random effect. NA indicates no detected individuals.

**Table 4:** Regression models to examine the potential differences in odds for detection of Cs-137 and Cs-134 before April 2013 between those who participated in the WBC monitoring and those who did not after April.

Variables	Cs-137 before April 2013	Cs-134 before April 2013
Whole body counter (WBC) monitoring participation after April 2013		
No	1.02 (0.92–1.13)	1.11 (1.00–1.23)
Yes	1.00	1.00
Age group (year)		
21–30	0.59 (0.48–0.72) ***	0.60 (0.49–0.74) ***
31–40	0.69 (0.59–0.81) ***	0.66 (0.56–0.78) ***
41–50	0.78 (0.66–0.91) **	0.75 (0.63–0.88) **
51–60	0.84 (0.73–0.97) *	0.88 (0.76–1.01)
61–70	1.00	1.00
71–80	1.25 (1.07–1.45) **	1.20 (1.02–1.40) *
81–	1.53 (1.19–1.97) **	1.18 (0.90–1.54)
Sex		
Men	1.00	1.00
Women	0.27 (0.24–0.31) ***	0.31 (0.27–0.35) ***
Height at WBC measurement	1.00 (0.99–1.01)	1.00 (0.99–1.01)
Weight at WBC measurement	1.02 (1.02–1.02) ***	1.02 (1.01–1.02) ***
Post-incident actual lived-at address		
Inside Minamisoma City	1.00	1.00
Outside Minamisoma City but inside Fukushima Prefecture	1.07 (0.93–1.24)	1.14 (0.99–1.32)
Neighboring prefectures of Fukushima	0.61 (0.50–0.74) ***	0.57 (0.46–0.70) ***
Outside the neighboring prefectures	0.31 (0.22–0.45) ***	0.30 (0.20–0.43) ***
Air dose rate (μSv/h) as of 29 April 2011 at pre-incident residential address	1.25 (1.17–1.34) ***	1.27 (1.21–1.34) ***

Note: * *p* < 0.05; ** *p* < 0.01; *** *p* < 0.001. Variables in the tables were mutually adjusted. Pre-incident residential address was also adjusted as a random effect. NA indicates no detected individuals.

## Supplementary Materials

The following are available online at www.mdpi.com/1660-4601/14/6/656/s1, Table S1. Regression models adopted to project the probability of Cs detection in the whole population (odds ratio and 95% CI) for Cs-137 (the upper) and Cs-134 (the lower).
