# Supplementary material for: Towards a Long-Term Strategy for Voluntary-Based Internal Radiation Contamination Monitoring: Representativeness of the Monitoring Results in Fukushima, Japan
Source: Int J Environ Res Public Health. 2017 Jun 20;14(6):656. doi: 10.3390/ijerph14060656 (PMC5486342; doi:10.3390/ijerph14060656)
Supplement: Supplementary file 1 [file ijerph-14-00656-s001.pdf]

Supplementary Materials: Towards a Long-Term Strategy for Voluntary-Based Internal Radiation Contamination Monitoring: Representativeness of the Monitoring Results in Fukushima, Japan

Table S1. Regression models adopted to project the probability of Cs detection in whole population (odds ratio and 95% CI) for Cs-137 (the upper) and Cs-134 (the lower).

Cs-137						
**Time Period**	**Oct 2011**	**Nov 2011**	**Dec 2011**	**Jan 2012**	**Feb 2012**	**Mar 2012 to Apr 2012**
Age group						
21–30	0.66 (0.39–1.13)	0.55 (0.34–0.89)*	0.54 (0.33–0.90)*	0.53 (0.27–1.05)	0.39 (0.20–0.76)**	0.33 (0.14–0.75)**
31–40	0.80 (0.50–1.27)	0.58 (0.40–0.85)**	0.53 (0.36–0.78)**	0.81 (0.46–1.41)	0.47 (0.25–0.85)*	0.30 (0.16–0.56)***
41–50	0.72 (0.45–1.15)	0.68 (0.46–0.98)*	0.62 (0.42–0.91)*	0.71 (0.40–1.26)	0.49 (0.27–0.88)*	0.84 (0.48–1.48)
51–60	0.94 (0.61–1.43)	1.01 (0.69–1.48)	0.76 (0.51–1.11)	0.83 (0.48–1.46)	0.69 (0.40–1.17)	0.85 (0.50–1.43)
61–70	1.00	1.00	1.00	1.00	1.00	1.00
71–80	1.25 (0.77–2.05)	1.11 (0.71–1.73)	1.21 (0.77–1.91)	2.45 (1.28–4.70)**	0.61 (0.28–1.29)	1.40 (0.79–2.47)
81–	1.12 (0.55–2.29)	1.60 (0.75–3.41)	1.58 (0.72–3.47)	1.48 (0.50–4.40)	0.20 (0.02–1.77)	0.60 (0.16–2.28)
Gender						
Men	1.00	1.00	1.00	1.00	1.00	1.00
Women	0.14 (0.11–0.19)***	0.18 (0.14–0.23)***	0.18 (0.14–0.23)***	0.11 (0.07–0.16)***	0.12 (0.08–0.17)***	0.17 (0.11–0.25)***
Post-incident actual lived-at address						
Inside Minamisoma City	1.00	1.00	1.00	1.00	1.00	1.00
Outside Minamisoma City but inside Fukushima Prefecture	0.97 (0.69–1.36)	0.96 (0.69–1.32)	1.28 (0.86–1.90)	0.58 (0.33–1.01)	0.89 (0.58–1.38)	1.51 (0.91–2.52)
Neighboring prefectures of Fukushima	0.51 (0.31–0.83)**	0.73 (0.47–1.13)	0.34 (0.19–0.62)***	0.61 (0.34–1.11)	0.57 (0.32–1.03)	0.65 (0.35–1.22)
Outside the neighboring prefectures	0.71 (0.26–1.92)	0.28 (0.09–0.83)*	0.54 (0.16–1.79)	0.41 (0.16–1.05)	0.19 (0.06–0.56)**	0.36 (0.12–1.10)
Air dose rate (μSv/h) as of 29 April 2011 at pre-incident residential address	1.05 (0.97–1.13)	1.30 (1.23–1.37)***	1.15 (1.00–1.32)	1.24 (1.10–1.38)***	1.15 (1.07–1.24)***	1.16 (1.04–1.28)**

**Time Period**	**May 2012**	**Jun 2012**	**Jul 2012 to Aug 2012**	**Sep 2012 to Mar 2013**	**Apr 2013 to Mar 2015**
Age group					
21–30	0.20 (0.06–0.71)*	0.29 (0.07–1.29)	0.06 (0.01–0.41)**	0.53 (0.24–1.16)	0.27 (0.08–0.87)*
31–40	0.32 (0.17–0.62)**	0.70 (0.31–1.58)	0.47 (0.25–0.88)*	0.33 (0.16–0.68)**	0.23 (0.09–0.57)**
41–50	0.44 (0.22–0.89)*	0.78 (0.35–1.74)	0.58 (0.32–1.03)	0.24 (0.11–0.54)**	0.33 (0.16–0.69)**
51–60	0.61 (0.35–1.06)	0.74 (0.38–1.43)	0.90 (0.58–1.40)	0.56 (0.34–0.91)*	0.41 (0.24–0.71)**
61–70	1.00	1.00	1.00	1.00	1.00
71–80	0.94 (0.57–1.55)	1.52 (0.87–2.65)	1.30 (0.84–2.01)	1.50 (0.97–2.30)	1.54 (1.07–2.20)*
81–	0.34 (0.10–1.22)	3.80 (1.60–9.01)**	1.64 (0.86–3.13)	2.91 (1.62–5.20)***	0.95 (0.49–1.83)
Gender					
Men	1.00	1.00	1.00	1.00	1.00
Women	0.13 (0.09–0.20)***	0.08 (0.05–0.14)***	0.12 (0.08–0.18)***	0.22 (0.15–0.31)***	0.32 (0.23–0.45)***
Post-incident actual lived-at address					
Inside Minamisoma City	1.00	1.00	1.00	1.00	1.00
Outside Minamisoma City but inside Fukushima Prefecture	0.23 (0.05–1.00)	0.69 (0.26–1.84)	0.63 (0.29–1.35)	0.79 (0.42–1.47)	0.78 (0.39–1.57)

Neighboring prefectures of Fukushima	0.57 (0.21–1.54)	0.56 (0.16–1.94)	0.88 (0.37–2.12)	0.20 (0.05–0.81)*	0.11 (0.02–0.82)*
Outside the neighboring prefectures	0.21 (0.03–1.64)	1.05 (0.22–5.06)	0.27 (0.04–2.03)	0.37 (0.09–1.53)	NA
Air dose rate (μSv/h) as of 29 April 2011 at pre-incident residential address	1.19 (0.90–1.57)	1.13 (0.93–1.36)	1.14 (1.01–1.28)*	1.14 (1.04–1.26)**	1.23 (1.16–1.31)***

Cs-134

**Time period**	**Oct 2011**	**Nov 2011**	**Dec 2011**	**Jan 2012**	**Feb 2012**	**Mar 2012 to Apr 2012**
Age group						
21–30	0.67 (0.38–1.17)	0.49 (0.30–0.80)**	0.53 (0.33–0.87)*	0.38 (0.18–0.81)*	0.31 (0.15–0.67)**	0.22 (0.08–0.59)**
31–40	0.80 (0.50–1.29)	0.42 (0.28–0.62)***	0.57 (0.39–0.83)**	0.62 (0.34–1.12)	0.43 (0.22–0.84)*	0.28 (0.14–0.56)***
41–50	0.56 (0.35–0.89)*	0.59 (0.40–0.86)**	0.60 (0.41–0.87)**	0.65 (0.36–1.19)	0.33 (0.16–0.66)**	0.39 (0.20–0.79)**
51–60	1.06 (0.69–1.63)	0.90 (0.61–1.33)	0.77 (0.53–1.12)	0.94 (0.53–1.68)	0.57 (0.31–1.05)	0.61 (0.35–1.09)
61–70	1.00	1.00	1.00	1.00	1.00	1.00
71–80	1.26 (0.77–2.06)	0.89 (0.57–1.41)	1.52 (0.97–2.37)	2.03 (1.04–3.98)*	0.48 (0.20–1.17)	1.12 (0.61–2.03)
81–	1.12 (0.55–2.28)	1.23 (0.57–2.67)	1.05 (0.48–2.32)	1.60 (0.53–4.77)	0.31 (0.03–3.19)	0.43 (0.09–2.01)
Gender						
Men	1.00	1.00	1.00	1.00	1.00	1.00
Women	0.12 (0.09–0.17)***	0.17 (0.13–0.22)***	0.19 (0.15–0.25)***	0.11 (0.07–0.16)***	0.09 (0.05–0.15)***	0.14 (0.09–0.23)***
Post-incident actual lived-at address						
Inside Minamisoma City	1.00	1.00	1.00	1.00	1.00	1.00
Outside Minamisoma City but inside Fukushima Prefecture	1.09 (0.76–1.56)	1.11 (0.80–1.54)	0.74 (0.50–1.11)	0.61 (0.34–1.11)	1.09 (0.66–1.78)	1.74 (0.99–3.06)
Neighboring prefectures of Fukushima	0.59 (0.36–0.99)*	0.58 (0.36–0.94)*	0.26 (0.15–0.48)***	0.45 (0.23–0.90)*	0.72 (0.37–1.43)	0.44 (0.19–1.01)
Outside the neighboring prefectures	0.33 (0.12–0.97)*	0.34 (0.11–1.03)	0.56 (0.19–1.62)	0.29 (0.10–0.90)*	0.13 (0.03–0.58)**	0.26 (0.06–1.16)
Air dose rate (μSv/h) as of 29 April 2011 at pre-incident residential address	1.11 (1.00–1.22)*	1.30 (1.20–1.41)***	1.01 (0.87–1.17)	1.23 (1.09–1.39)**	1.19 (1.07–1.32)**	1.10 (0.98–1.23)

**Time period**	**May 2012**	**Jun 2012**	**Jul 2012 to Aug 2012**	**Sep 2012 to Mar 2013**	**Apr 2013 to Mar 2015**
Age group					
21–30	0.11 (0.01–0.85)*	0.25 (0.03–1.94)	0.44 (0.17–1.14)	0.10 (0.01–0.77)*	0.23 (0.03–1.66)
31–40	0.26 (0.11–0.61)**	0.52 (0.17–1.58)	0.10 (0.02–0.40)**	0.36 (0.15–0.87)*	0.23 (0.05–0.97)*
41–50	0.31 (0.12–0.78)*	0.53 (0.18–1.61)	0.63 (0.33–1.20)	0.35 (0.14–0.85)*	0.10 (0.01–0.74)*
51–60	0.49 (0.25–0.96)*	0.95 (0.45–2.00)	0.96 (0.59–1.58)	0.59 (0.32–1.07)	0.19 (0.06–0.62)**
61–70	1.00	1.00	1.00	1.00	1.00
71–80	1.01 (0.57–1.79)	1.40 (0.72–2.70)	1.45 (0.90–2.34)	1.62 (0.96–2.72)	1.39 (0.80–2.41)
81–	NA	2.52 (0.90–7.06)	1.50 (0.72–3.12)	2.02 (0.95–4.29)	0.77 (0.27–2.25)
Gender					
Men	1.00	1.00	1.00	1.00	1.00
Women	0.16 (0.09–0.27)***	0.09 (0.04–0.18)***	0.25 (0.17–0.37)***	0.49 (0.32–0.73)**	0.31 (0.18–0.54)***
Post-incident actual lived-at address					
Inside Minamisoma City	1.00	1.00	1.00	1.00	1.00
Outside Minamisoma City but inside Fukushima Prefecture	0.55 (0.12–2.49)	0.44 (0.10–1.89)	0.58 (0.23–1.46)	0.95 (0.44–2.02)	NA
Neighboring prefectures of Fukushima	0.20 (0.03–1.48)	0.32 (0.04–2.45)	1.06 (0.42–2.69)	0.82 (0.29–2.33)	NA
Outside the neighboring prefectures	0.42 (0.05–3.31)	0.93 (0.11–7.51)	0.79 (0.19–3.37)	1.06 (0.32–3.52)	NA
Air dose rate (μSv/h) as of 29 April 2011 at pre-incident residential address	1.11 (0.81–1.52)	1.05 (0.83–1.34)	1.16 (1.04–1.29)**	1.21 (1.09–1.34)***	1.23 (1.12–1.36)***

Note: * p<0.05, **<0.01, ***<0.001. Variables in the tables were mutually adjusted. Pre-incident residential address was also adjusted as a random effect. NA indicates no detected individuals.
